# Diphtheria's resurgence and the high cost of misinformation

**DOI:** 10.1016/j.ebiom.2025.105892

**Published:** 2025-08-13

**Authors:** eBioMedicine

In this issue of *eBioMedicine*, Abbas and colleagues report on a 2022–23 diphtheria outbreak of more than 18,000 cases in Kano, Nigeria, following vaccination campaign disruptions during the COVID-19 pandemic. The case fatality rate was estimated to be 4.5%, with unvaccinated individuals more than twice as likely to be at risk of death than fully vaccinated individuals (AOR 2.45; 95% CI 2.05–2.94, p < 0.0001).

The resurgence of diphtheria in Kano highlights the persistent threat posed by this once common childhood disease. Despite decades of progress achieved through vaccination, disruptions in immunisation programmes—exacerbated by a rise in vaccine misinformation—threaten to leave communities susceptible again.

Until the advent and wide availability of the first diphtheria vaccines in early 20th century, this disease was a common cause of death globally, especially among children. Sometimes referred to as “the strangling angel of children”, diphtheria is a highly contagious disease caused by the pathogenic, toxin-producing form of the bacterium *Corynebacterium diphtheriae*. Upon infection, release of bacterial diphtheria toxin into the host cell leads to cellular death and subsequent necrosis of affected tissue. In respiratory cases, formation of a thick, so-called pseudomembrane can occur as the disease progresses, leading to a swelling of the neck and blocking of airways, making it difficult for the patient to swallow or take a full breath. The bacterial toxin can also spread from the airways to the heart, kidney, and nervous system, and severe cases can be fatal, even with treatment such as diphtheria antitoxin and antibiotics.

Early vaccines against diphtheria were based on formalin-inactivated diphtheria toxin (toxoid), plus aluminium salt, which was included as an adjuvant to enhance the immune response. Later, in the 1940s, diphtheria toxoid was combined with tetanus toxoid and whole-cell, inactivated *Bordatella pertussis* (DTwP), to create the first diphtheria–tetanus–pertussis (DTP) vaccines. Combination DTP formulations can now also include additional vaccines against hepatitis B virus, Haemophilus influenzae, or poliovirus, depending on regional recommendations.

In part to address concerns related to the potential for increased reactogenicity of the whole cell pertussis-causing bacterium, an acellular form of the pertussis component (DTaP) was introduced in the 1990s and is now the most widely used formulation in high-income regions such as the USA and Europe. However, many resource-challenged regions around the world continue to use whole cell as the pertussis vaccine component of choice. Although whole-cell combination vaccines are associated with a higher incidence of common side effects (eg, fever and local reactions at the injection site) than acellular–vaccine combinations, DTwP vaccines are favoured in some low-income regions due to their lower cost than DTaP, and because fewer doses are needed for effective prevention of severe disease and mortality.

Studies in non-human primates have also shown that whole-cell-vaccinated animals cleared *B pertussis* challenge infections more quickly than acellular-vaccinated animals, and that they were able to mount protective T helper-17 cell and T helper-1 cell-mediated responses, which have been shown in mice to be important for long-term immune memory to *B pertussis*. A 2024 study has also confirmed in humans that the frequency of interleukin-17 producing tissue-resident memory T cells was significantly higher in adults who had received whole-cell-containing vaccines as children—compared with those who had received acellular-containing vaccines—indicating the protective immune response offered by the whole-cell vaccine is long-lived, potentially lasting decades.

Despite proven benefits and practical advantages, DTwP vaccines have not been free from controversy over the years. In 1982, a documentary entitled, “DPT: Vaccine Roulette” falsely claimed that the pertussis component of the vaccine caused encephalopathy and neurological complications in vaccinated children, based in part on an interpretation of an uncontrolled case series published in 1974. Several larger, well-controlled studies such as a New England Journal of Medicine paper published in 2001 have since refuted these claims and have definitively shown that, although febrile seizures were more common in those receiving DTwP vaccines, children who had febrile seizures from vaccination were neither at increased risk for long-term neurodevelopmental disability nor for later seizures.

However, the damage had been done. In the 1970s and 1980s, anti-vaccine movements questioning DTwP safety began to take hold, spreading misinformation and leading to widespread immunisation disruptions in countries including Sweden, Japan, and the UK. In 1998, a study in *The Lancet* found that in countries where anti-DTP vaccination movements negatively affected vaccination coverage, pertussis incidence rose to rates 10–100 times higher than countries where high DTP vaccination coverage had been maintained throughout the same time period. Like diphtheria, pertussis can be a devastating disease in children—especially in infants younger than 1 year.

With the 2022–23 outbreak of diphtheria cases in Kano, it is more important than ever that adequate vaccination coverage is achieved in susceptible regions. GAVI, in partnership with WHO (funded in part by government donors such as the UK and the USA and by philanthropic institutes such as the Bill and Melinda Gates Foundation), is at the forefront of efforts to ensure that children in low-income countries have access to life-saving vaccines. A recent announcement by the US Health Secretary, Robert F Kennedy Jr, that the USA would not deliver on its promise of $1.2 billion in government funding to GAVI, therefore comes as a devastating blow with terrible timing. Kennedy, a long-time vaccine sceptic, cited concerns about DTwP safety as part of the rationale for withdrawing US funding, and referenced a 2017 observational *eBioMedicine* study from Peter Aaby and colleagues, in which the authors had examined non-specific effects of the vaccine on all-cause mortality, using retrospective data obtained in Guinea-Bissau from the 1980s. The work referenced by Kennedy was one of several observational studies published in 2016–18 from the Aaby group, which had identified what the researchers suggested was a potential signal linking non-specific effects of the vaccine with an increase in overall mortality, particularly in girls. However, a 2022 follow-up analysis from the same group, which assessed more recent data from 2010 to 14 (also from Guinea-Bissau), found no such link between the vaccine and all-cause mortality.

An estimated 41,463,000 (95% CI 39,903,000–42,706,000) lives have been saved since 1972 due to the administration of safe and effective combination vaccines against diphtheria, pertussis, and tetanus—three childhood diseases once considered common around the globe. Based on ongoing and independent assessment of several decades of published data by WHO's Strategic Advisory Group of Experts, any decisions made by GAVI regarding vaccine investment are made based on the best available science. To preserve the extraordinary gains made against preventable childhood diseases, policy makers must prioritise scientific consensus over misinformation and ensure sustained support for vaccination initiatives.

